# Effect of interval compared to continuous exercise training on physiological responses in patients with chronic respiratory diseases: A systematic review and meta-analysis

**DOI:** 10.1177/14799731211041506

**Published:** 2021-10-19

**Authors:** Charikleia Alexiou, Lesley Ward, Emily Hume, Matthew Armstrong, Mick Wilkinson, Ioannis Vogiatzis

**Affiliations:** 1 Department of Sport, Exercise and Rehabilitation, Faculty of Health and Life Sciences, 373117 Northumbria University Newcastle, Newcastle Upon Tyne, UK

**Keywords:** exercise, respiratory disease, systematic review

## Abstract

Current evidence suggests that interval exercise training (IET) and continuous exercise training (CET) produce comparable benefits in exercise capacity, cardiorespiratory fitness and symptoms in patients with chronic obstructive pulmonary disease (COPD). However, the effects of these modalities have only been reviewed in patients with COPD. This meta-analysis compares the effectiveness of IET versus CET on exercise capacity, cardiorespiratory fitness and exertional symptoms in patients with chronic respiratory diseases (CRDs). **Methods:** PubMed, CINHAL, Scopus, Cochrane Central Register of Controlled Trials (CENTRAL) and Nursing and Allied health were searched for randomised controlled trials from inception to September 2020. Eligible studies included the comparison between IET and CET, reporting measures of exercise capacity, cardiorespiratory fitness and symptoms in individuals with CRDs. **Results:** Thirteen randomised control trials (530 patients with CRDs) with fair to good quality on the PEDro scale were included. Eleven studies involved *n* = 446 patients with COPD, one involved *n* = 24 patients with cystic fibrosis (CF) and one *n* = 60 lung transplantation (LT) candidates. IET resulted in greater improvements in peak work rate (WR_peak_) (2.40 W, 95% CI: 0.83 to 3.97 W; *p* = 0.003) and lower exercise-induced dyspnoea (−0.47, 95% CI: −0.86 to 0.09; *p* = 0.02) compared to CET; however, these improvements did not exceed the minimal important difference for these outcomes. No significant differences in peak values for oxygen uptake (VO_2peak_), heart rate (HR_peak_), minute ventilation (VE_peak_), lactate threshold (LAT) and leg discomfort were found between the interventions. **Conclusions:** IET is superior to CET in improving exercise capacity and exercise-induced dyspnoea sensations in patients with CRDs; however, the extent of the clinical benefit is not considered clinically meaningful.

## Introduction

In patients with chronic respiratory diseases (CRDs), exercise intolerance, the inability to undertake exercise at the work rate expected for a healthy age‐matched individual, is common.^1^ Regardless of the type of CRD, this inability is commonly caused by impairment of several physiological systems and associated with the intensification of breathlessness and peripheral muscle discomfort.

Exercise training aims to improve cardiorespiratory fitness and condition the muscles of ambulation, to increase exercise tolerance and reduce breathlessness and leg discomfort. To obtain improvements in exercise tolerance, an increased volume of moderately intense continuous exercise is recommended.^2^ However, patients with profound ventilatory limitation are unable to sustain moderately intense exercise for sufficiently long periods to induce significant physiological adaptations when continuous exercise modalities are implemented.^3^ This is primarily due to intense breathlessness compromising exercise tolerance. In these patients, high intensity interval exercise training (IET), consisting of repeated bouts of maximal/high intensity exercise, alternated with short intervals of rest or low intensity exercise, constitutes a suitable alternative to continuous exercise training (CET).^4^

A meta-analysis comparing the effect of IET and CET on cardiorespiratory, functional capacity and health-related quality of life (HRQoL) in patients with chronic obstructive pulmonary disease (COPD), concluded that IET was as effective as CET in improving these outcomes.^5^ A subsequent Cochrane review^6^ examining the optimal intensity of exercise training in COPD patients concluded that high intensity IET was equally effective to moderate CET in improving exercise capacity, symptoms and HRQoL. Similarly, a recent narrative review^7^ concluded that IET produces similar changes in cardiorespiratory fitness and exercise capacity as CET in COPD patients, while noting a paucity of studies exploring the effects of IET in other types of CRDs.

This study extends previous findings by assessing the effectiveness of IET compared to CET across a range of CRDs (COPD, cystic fibrosis (CF) and lung transplant (LT) candidates). Outcome variables investigated include physiological responses (1) at peak exercise (work rate, oxygen uptake, minute ventilation and heart rate), (2) during submaximal levels of incremental exercise (anaerobic threshold) and (3) peak sensations of dyspnoea and leg discomfort. We aimed to investigate whether IET is superior to CET in improving exercise capacity and physiological responses in patients with CRDs.

## Methods

### Protocol registration

This systematic review and meta-analysis was undertaken following the Preferred Reporting Items for Systematic Reviews and Meta-Analyses (PRISMA) guidelines^8^ and the Cochrane Handbook for Systematic Reviews of Interventions^9^ and is registered with the International Prospective Register of Systematic Reviews (CRD42021173562).

### Search strategy and screening

A comprehensive search was conducted in clinically relevant databases: PubMed, CINHAL, Scopus, Cochrane Central Register of Controlled Trials (CENTRAL) and Nursing and Allied Health. Databases were searched from inception to September 2020. Search strategies were developed and piloted in consultation with a librarian, with reference to previous reviews.^5,6^ Search strategies used MeSH terms combined with keywords related to the target population (‘lung diseases’), intervention (‘interval’, ‘intermittent’ and ‘high intensity’), comparator (‘exercise’, ‘rehabilitation’, ‘continuous’, ‘moderate intensity’, ‘aerobic’ and ‘endurance’) and outcomes (‘exercise tolerance’, ‘exercise capacity’, ‘dyspnea’, ‘leg fatigue’ and ‘quality of life’). Searches were limited to English language articles published in peer-reviewed journals. Additionally, reference lists of included studies and related reviews were hand searched to identify any eligible studies. The completed PubMed search strategy is presented in Table S1 (online supplement). Two authors (CA and EH) independently screened titles, abstracts and full texts of retrieved articles, with any disagreements resolved through discussion with a third author (MA).

### Inclusion criteria

Study eligibility was pre-determined according to the following inclusion criteria:

*Participants:* Adults aged > 18 years old, diagnosed with one of the following CRDs: COPD, CF, bronchiectasis, asthma, pulmonary arterial hypertension, interstitial lung disease and lung transplantation candidates.
*Intervention:* Studies comparing the effectiveness of IET versus CET incorporated into pulmonary rehabilitation.^2^ IET consisted of repeated brief bouts of high intensity exercise, alternated with either passive or low-moderate intensity recovery periods on treadmill or cycle ergometer. CET consisted of constant-load exercise on treadmill or cycle ergometer sustained at moderate intensities.
*Outcome Measures:* Peak work rate (WR_peak_), peak oxygen uptake (VO_2peak_), peak minute ventilation (VE_peak_), peak heart rate (HR_peak_), oxygen uptake at the lactate threshold (LAT), modified Borg’s scale (CR-10)^10^ for dyspnoea and leg discomfort assessed through incremental cardiopulmonary exercise testing. Accordingly, we focused on the effect of IET versus CET on physiological variables recorded during cardiopulmonary exercise testing, to justify potential differences in WR_peak_, in conjunction with differences in the magnitude of physiological adaptations.
*Study Design*: Randomised control trials (RCTs)

### Data extraction

Study characteristics and outcome data were extracted relating to article information (first author, year of publication), participant characteristics (age, gender and lung function) and study design and setting, intervention parameters and outcome measures (exercise capacity, cardiorespiratory fitness and symptoms) (Table 1).

**Table 1.** Characteristics of included randomised controlled studies.

Author, Year	CRD	Setting	Sample	Age (year),	FEV1 (L)	Intervention	Comparator	Duration (wks)	Outcomes
			Size (*n*)	Gender(M/F)	(% pred)	(IET) parameters	(CET) parameters	Frequency (times × per wk)	1) Exercise capacity2) Cardiorespiratory fitness3) Symptoms (dyspnoea and leg discomfort)
Coppoolse^18^	COPD	Netherlands, supervised inpatient programme	21	62 year for IET, 67 year for CET, 100%M	37±15%	60 s (90% WR peak) cycling alternating with 120 s (45% WR peak) for 30 min	Cycling (65% WR peak) for 30 min	8 wks, 3 × wk for IET, 2 × wk for CET	1) Incremental and CWR: WR_peak_ and VO_2__peak_ 2) VE_peak_, HR_peak _and LAT 3) Visual analogue scale (VAS)
Vogiatzis^4^	COPD	Greece, supervised outpatient PR	36	68 year, 83%M	45 ± 4%	30 s cycling (100% WR peak) alternating with 30 s rest period for 20 min per session, 40 min total	Cycling (50% WR peak) for 40 min per session	12 wks, 2× wk, education, instruction in the use of medication, dietary advice, psychological Support, breathing exercises	1) Ramp incremental: WR_peak_ and VO_2__peak_ 2) VE_peak_, HR_peak _and LAT 3) Borg’s scale (0–10) for dyspnoea
Vogiatzis^16^	COPD	Greece, supervised outpatient PR	19	65 year, 84%M	40 ± 4%	30 s cycling (100% WR peak) alternating with 30 s rest period for 45 min per session	Cycling (60% WR peak) for 30 min	10 wks, 3 × wk, plus breathing, education, relaxation, secretion clearance techniques, Psychosocial support, progression monthly equal in magnitude in both groups	1) Incremental: WR_peak _and VO_2__peak_, 2) VE_peak_, HR_peak _and LAT 3) Modified Borg’s scale
Puhan^19^	COPD	Switzerland, supervised inpatient PR	86	69 year, 29% M for IET, 36%M for CET	34%	20 s cycling (100% WR peak) alternating with 40 s (20 % WR peak) for 20 min session	Cycling (60% WR peak) for 20 min	3 × wks (included 12 to 15 sessions), 4 × wk, 24 min session, total duration 5 weeks of PR, progression: WR increased by 10% of baseline for each group	1) Incremental, steep ramp test: WR_peak_ 2) Not assessed 3) CRQ - all domains
Arnardottir^23^	COPD	Sweden, supervised outpatient PR	60	43 to 80 year, 13%M	14% To 59%	180 s cycling (80% WR peak) alternating with 180 s (40% WR peak) for 27 min	Cycling (65% WR peak) for 27 min	16 wks, 2 × wk (1 day/week) calisthenics, 1 day/week resistance training and relaxation, progression performed but not explicitly explained	1) Incremental cycle test, semi-steady state cardiopulmonary test: WR_peak_ and VO_2__peak_ 2) VE_peak_ and HR_peak_ 3) Borg dyspnoea (CR-10) and RPE
Varga^22^	COPD	Hungary, supervised outpatient PR	79 (only 39 randomised in groups)	64 year, 77%M	57%	120 s (90% WR peak) cycling alternating with 60 s active rest (50% WR peak) for 30 min	Cycling (80% WR peak) for 30 min or self-paced training: Unsupervised self-paced group on cycle, walk and climb stairs in own environment	8 wks, 3 × wk, 45 min per session, progression of exercise duration targeted to 45 min, but proportion and Frequency of progression not explicitly explained	1) Incremental: PWR and VO_2peak_ 2) VE_peak_, HR_peak_ and LAT 3) Borg (CR-10) scores of dyspnoea and RPE
Mador^24^	COPD	New York, Veterans Affairs New York healthcare, supervised outpatient PR	41	72 year, 80%M	45%	Cycling and treadmill: 60 s (150% WR peak) alternating with 120 s (75% WR peak) for 21 min, total training duration 42 min/session	Cycling (50% WR peak) for 20 min and treadmill at 80% of average speed in 6MWT and 0% elevation (20 min), total training duration 40 min	Cycle and treadmill: 8 wks, 3 × wk, stretching, calisthenics, 1-h/wk education, 21 min duration, 24 sessions, progression in both groups: Cycling WR increased by 10% and treadmill speed increased by 5%–10%	1) Incremental: WR_peak _and VO_2__peak_ 2) VE_peak _and HR_peak_ 3) CRQ - all domains
Nasis^21^	COPD	Greece, supervised outpatient PR	42	66 year, 78%M	42%	30 s cycling at (100% WR peak) alternated with 30 s rest for 40 min per session	Cycling (60% WR peak) for 30 min per session	10 wks, 3 × wk plus education and breathing exercises weekly increase of total work rate for each group not explicitly explained	1) Incremental: WR peak 3) Borg (CR-10) dyspnoea and RPE (leg fatigue)
Gloeckl^17^	LT candidates	Germany, supervised inpatient PR	60	53 ± 6 year, 47%M	25 ± 8%	30 s cycling alternating with 30 s rest. Resistance exercises	Cycling (60 % WR peak), resistance exercises	3 × wks, 5–6 × wk	1) Incremental: WR_peak_ 2) Not assessed 3) Modified Borg’s scale (0–10)
Bronstad^26^	COPD	Norway, supervised outpatient PR	20	64 year, 70%M	52,8 ± 11%	Uphill treadmill walking: 90% HR peak, 38 min (intervals of 4 min: 4 min)	Treadmill uphill walking (70% HR peak) for 47 min	10 wks, 3 × wk	1) Ramp protocol: VO_2__peak_ 2) VE_peak_, HR_peak _and LAT 3) Not assessed
Rizk^20^	COPD	Canada, supervised inpatient programme	35	68 year, 35%M	60.2 ± 15.8%	30 s intervals at the 100% WR peak alternating with 30 s	Cycling HR (equivalent to HR reached at 80% WR peak) for 25 min	12 wks, 3 × wk, 3 groups of exercise, continuous, high intensity, continuous ventilatory threshold, interval. 10 min warm up (5 min unloaded pedalling + 5 min progressively increasing load) + 5 min cool down	1) Incremental: WR peak and VO_2__peak_ 2) VE_peak_ and HR_peak_ 3) Borg’s scale (0–10)
Kaltsakas^15^	CF	Greece, structured, outpatient hospital-based PR programme	24	32 year, 54%M	46%	30 min high intensity IET (100% WR peak for 30 s alternated with 40% WR peak for 30 s	Cycling (70% WR peak) for 30 min	12 wks, 3 × wk	1) Incremental: WR_peak_ and VO_2__peak_ 2) VE_peak _and HR_peak_ 3 Modified Borg’s scale
Ercin^25^	COPD	Turkey, supervised outpatient PR	69	60 year, 78%	55 ± 11%	wk 1–2: Cycling for 30 s at 100% WR peak alternating with 30 s rest, wk 3–8: 30 s at 120 % WR peak progressively to 140% WR peak alternating with 30 s rest	wk 1–2: 50% WR peak, wk 3–4: 60% WR peak, wk 5–8: 70% WR peak cycling for 3 min	8 × wks, 3 × wk, 30 min sessions	1) Incremental: WR_peak_ and VO_2__peak_ 2) VE_peak_ and HR_peak_ 3) Borg’s scale

F: female, M: male, IET: Interval exercise training, CET: continuous exercise training, wks: weeks, CWR: constant work rate, WR peak: peak work rate CRQ: chronic respiratory disease questionnaire, RPE: ratings for perceived exertion, LAT: lactate threshold, PR: pulmonary rehabilitation programme, RCT: randomised control trial.

Outcome data related to mean difference (pre-training to post-training) and standard deviation were extracted. If pre-training values were lacking, baseline values obtained from the symptom limited incremental exercise protocol^5,6^ were used. Any missing data were imputed from other reviews.^5,6^ To determine the magnitude and clinical benefit for each outcome, we compared the treatment effect and 95% confidence interval (CI) with the minimal important difference (MID).

### Data synthesis

A meta-analysis comparing the different training types included in the studies was conducted using review manager.^11^ The difference in pre- to post-training change between IET and CET was calculated (IET minus CET) for each study. Treatment effects between studies were anticipated to vary; hence, meta-analysis was undertaken using the random-effects model. The random-effects model involves the extent of heterogeneity between study variations. Pooled effect sizes were expressed as mean difference (MD) between training groups with 95% CIs, with a threshold of *p* < 0.05 considered significant.

For outcomes where improvement was indicated by an increased outcome value, a positive MD represented a beneficial effect of IET over CET and a negative MD favoured CET. Conversely, where improvement in outcome measures was indicated by a decreased score following training (LAT, symptoms), a negative MD favoured IET and a positive MD favoured CET. Forest plots were produced for each outcome to compare results across studies. Heterogeneity was assessed using the *Q* statistic and *I*^2^ statistic. If significant heterogeneity was noted (*I*^2^ >40%), subgroup analysis was performed to investigate the heterogeneous results. Subgroup analysis involved splitting studies as follows: Participants’ characteristics: BMI < 30 kg/m^2^ and BMI > 30 kg/m^2^^12^ since tailoring exercise prescription to patients’ needs and capabilities could importantly influence physiological responses.^13^

Assessment of publication bias using a funnel plot analysis was feasible when at least 10 studies showed outcome data.^12^ A triangular 95% confidence region based on a fixed-effect meta-analysis was included in the plot.^12^ The funnel plot should be symmetrical in the absence of publication bias.^12^

Sensitivity analysis was used to examine whether the overall findings from the primary meta-analysis were robust to potentially influential decisions. Sensitivity analysis was conducted if there was evidence of poor to fair quality according to the PEDro scale score, indicating high or unclear risk of bias to treatment effects.^12^

## Results

### Quality assessment

Quality appraisal was independently assessed by two authors (CA and EH), using the Physiotherapy Evidence Database (PEDro) scale^14^ with discrepancies resolved by consensus. The PEDro scale^14^ assesses 11 items relating to allocation, baseline similarity, blinding, follow-up rates and analysis. Ten of the 11 items are scored as yes (1) or no (0), with summative scores indicative of excellent (9–10), good (6–8), fair (4–5) or poor (≤3) methodological quality.^14^ Trials were not excluded based on quality.

### Description of selected studies

**Figure 1.**
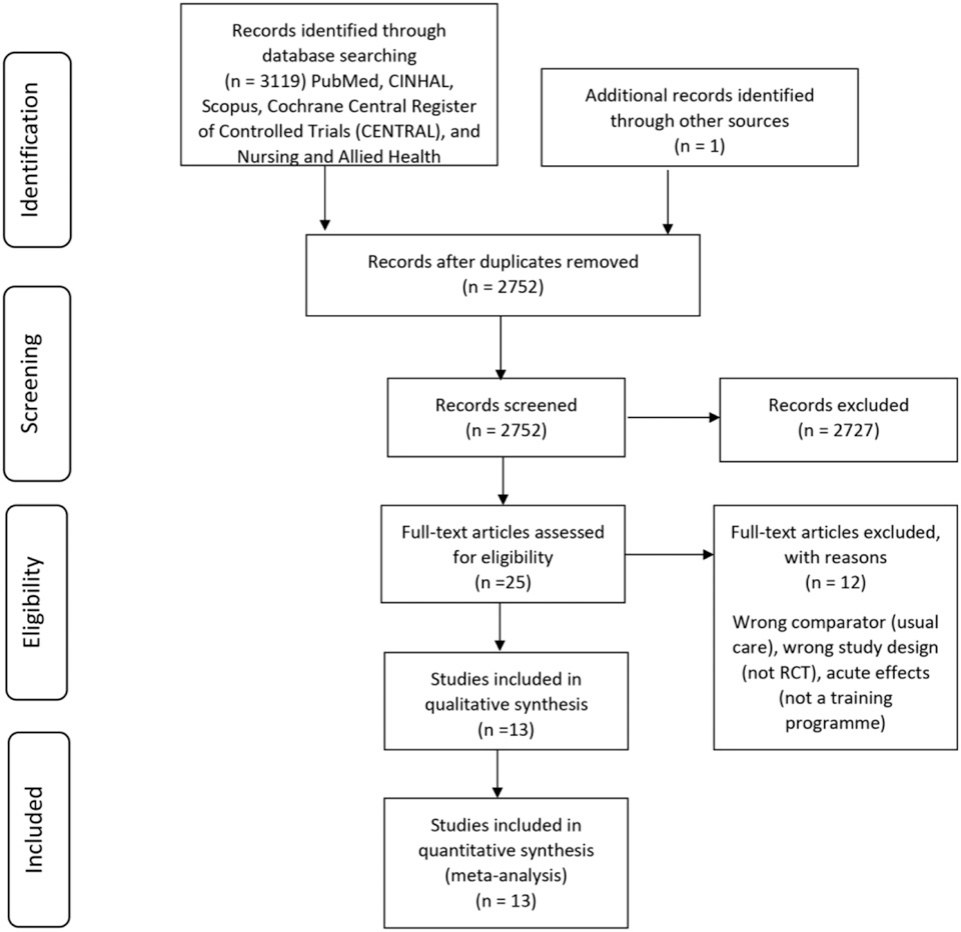
Search and selection of studies for systematic review according to preferred reporting items for systematic reviews and meta-analyses (PRISMA).

Searches generated 3119 studies (Figure 1); hand searching retrieved one additional article (conference abstract). Following removal of 368 duplicates, 2751 study title and abstracts were screened, 24 eligible studies underwent full-text screening, of which 12 studies met the inclusion criteria. Subsequent to the initial screening process, the conference abstract retrieved via hand searching became available as a full text version and has been included,^15^ making a total of 13 studies included in this review.

### Participant characteristics

A total of 530 patients with three CRDs were included in the 13 studies. Eleven studies^4,16-25^ were conducted in patients with COPD, one in patients with CF patients^15^ and one in LT candidates^17^ (Table 1).

Both exercise training modalities included a majority of men (61% in IET and 65% in CET) and elderly patients with mean age 65 ± 6 years for the IET and 62 ± 6 years for the CET group. Patients had been diagnosed with moderate to severe COPD (mean FEV_1_ % predicted: 43.6 ± 13.6% and FEV_1_/FVC: 50 ± 62%. Patients with CF had a mean FEV_1_ % predicted: 46 ± 22% and FEV_1_/FVC: 65 ± 115%. LT candidates had mean FEV_1_ % predicted: 25 ± 8% and FEV_1_/FVC: 35 ± 8%.

### Intervention characteristics

Four^18,19,20,17^ of the 13 studies included supervised inpatient rehabilitation and nine studies^4,15,16,18,21-26^ included supervised outpatient rehabilitation (Table 1). The prevailed training mode was cycle-based exercise followed by treadmill-based exercise.^24,26^ Programme duration ranged from 3 to 16 weeks, with eight weeks being the most common. Session frequency varied between two to six times weekly, with an average duration of 26 minutes for the IET and 30 minutes for the CET. Nine studies^4,15-18,21-25^ presented intensity of the training programmes as a fraction of WR_peak_ recorded during a symptom-limited incremental exercise test. One study^26^ calculated intensity as a fraction of HR_peak_ on the treadmill. The most widely used IET protocol consisted of alternating 30-s intervals at 100% WR_peak_ followed by 30-s active recovery (unloaded pedalling) on the cycle ergometer.^4,16,17,20,21,25^ Three studies^22,23,26^ applied longer intervals ranging from 2 to 4 min at 70–80% WR_peak_ alternating with 1–3 min at 40–70% WR_peak_ active recovery periods. Two studies^18,24^ implemented high intensity 1-min intervals at 90% WR_peak_ alternating with 2-min active recovery periods of low intensity at <75% WR_peak_. In one study,^19^ participants performed shorter intervals of 20 s at 100% WR_peak_ alternating with 40-s at 20% WR_peak_.

Total volume of work: Seven of the 13 studies^4,15,16,21,22,24^ reported equivalent total training work rate between IET and CET. Two studies^26,17^ reported that IET and CET protocols were matched in terms of equivalent energy expenditure. Four studies^19,20,23,25^ presented a tendency towards a lower total work in the IET group.

### Quality assessment

Overall, study quality was fair to good, with a mean PEDro score of six out of a possible 10 (range 5 to 8) (Table 2). Lower methodological quality was associated with inability to blind subjects or therapists, an inherent problem in training interventions. However, six of the 13 studies^15,^ reported blinded study assessors. Intention-to-treat analysis was reported in four studies.^17,19,22,24^

**Table 2.** PEDro quality assessment.

Author	Eligibility criteria	Random allocation	Concealed allocation	Baseline similarity	Blinding subject	Blinding therapist	Blinding assessor	Measure > 85% follow-up	ITT	Group statistical Comparison	Point/validity measure	Quality score	Quality
Coppoolse^18^	Y	Y	Y	Y	N	N	Y	Y	Y	Y	Y	8	Good
Vogiatzis^4^	Y	Y	N	Y	N	N	N	Y	N	Y	Y	5	Fair
Vogiatzis^16^ Puhan^19^ Arnardottir^23^	Y Y Y	Y Y Y	N Y Y	Y Y Y	N N N	N N N	N Y N	Y Y Y	N Y N	Y Y Y	Y Y Y	5 8 6	Fair Good Good
Varga^22^	Y	Y	N	Y	N	N	N	Y	Y	Y	Y	6	Good
Mador^24^	Y	Y	Y	Y	N	N	Y	Y	Y	Y	Y	8	Good
Nasis^21^	Y	Y	N	Y	N	N	N	Y	N	Y	Y	5	Fair
Gloeckl^17^	Y	Y	Y	Y	N	N	Y	Y	Y	Y	Y	8	Good
Bronstad^26^	Y	Y	Y	Y	N	N	Y	Y	N	Y	Y	7	Good
Rizk^20^	Y	Y	N	Y	N	N	Y	N	N	Y	Y	5	Fair
Kaltsakas^15^	Y	Y	Y	Y	N	N	Y	Y	N	Y	Y	7	Good
Ercin^25^	Y	Y	N	Y	N	N	N	Y	N	Y	Y	5	Fair

ITT: intention-to-treat, Y: yes, score = 1; N-no, score = 0. The higher the cumulative score, the higher the methodological quality as it follows: excellent (9–10), good (6–8), fair (4–5) and poor (<3). The score for eligibility criteria is not included in the cumulative score.

### Meta-analyses of included studies

Five indicators were used to assess the cardiorespiratory fitness of participants following completion of the IET and CET programmes: WR_peak_; VO_2peak_; HR_peak_; VE_peak_ and LAT.

**Figure 2.**
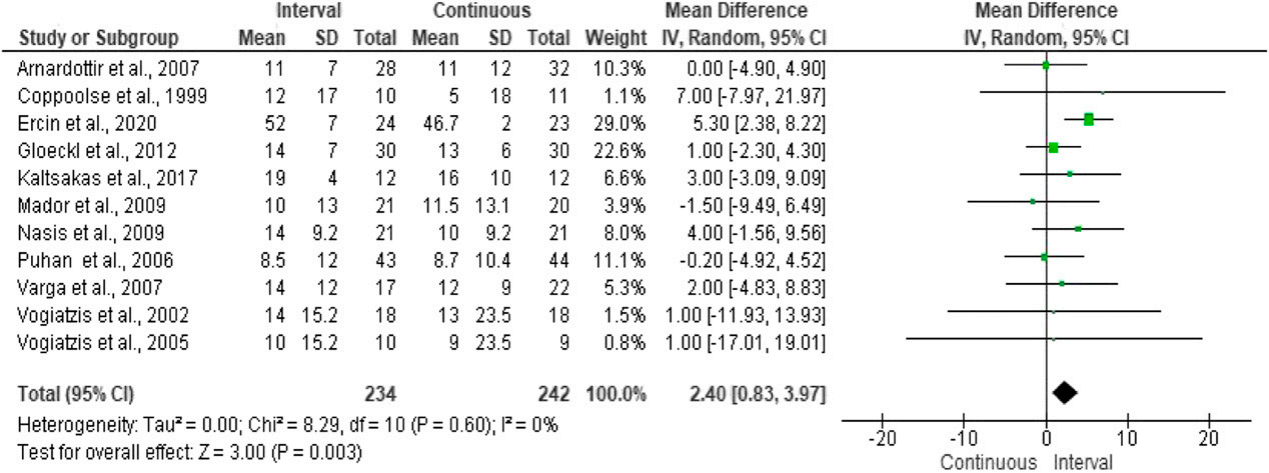
Comparison of the effect of IET versus CET on peak work rate (WR_peak_) in Watts.

The effect of the two training modalities on exercise capacity was reported in 11^4,15-19,21-25^ out of 13 studies using incremental cycle ergometry. Pooled results in the primary meta-analysis revealed a significant effect on WR_peak_ favouring IET compared to CET (MD = 2.40 W, 95% CI: 0.83–3.97 W; *p* = 0.003). No important heterogeneity was detected (*Q* = 8.29, *df* = 10, *I*^2^ = 0%; *p* = 0.60) (Figure 2), and as a result subgroup analysis was not performed. Funnel plot asymmetry suggested publication bias among studies as some studies were of lower methodological quality and therefore produced exaggerated intervention effect estimates. Sensitivity analysis influenced the direction of the treatment effect of the outcome, showing no difference between IET compared to CET in studies with participants with normal BMI.

VO_2peak_ (L/min) was assessed in nine studies.^4,15,16,18,22-26^ No significant effect was shown in the primary meta-analysis (MD = 0.01 L/min, 95% CI: −0.02 to 0.04 L/min; *p* = 0.55), indicating no difference in VO_2peak_ between training groups. No important heterogeneity was detected among studies (*Q* = 5.17, *df* = 8, *I*^2^ = 0%; *p* = 0.74) (Figure S1.1), and as a result, subgroup analysis was not performed for this outcome. Funnel plot was not feasible as less than ten studies were included in the meta-analysis. Sensitivity analysis did not affect the direction or significance of the treatment effect, suggesting no difference between training modalities.

Seven studies^4,15,22-26^ assessed HR_peak,_ presenting no significant differences between IET and CET (MD = 0.18 beats/min, 95% CI: −3.73 to 4.09 beats/min; *p* = 0.93) (Figure S1.2). However, considerable heterogeneity was identified among studies (*Q* = 167.15, *df* = 6, *I*^2^ = 96%; *p* = 0.00001). Significant subgroup differences were identified, suggesting that BMI had an influence on the treatment effect between subsets of studies. Publication bias was not assessed as less than ten studies were included in the meta-analysis of this outcome. Sensitivity analysis did not affect the direction or significance of the outcome.

VE_peak_ (L/min) was comparable among seven studies,^4,15,22-26^ with no significant difference between IET and CET (MD = 0.82 L/min, 95% CI: −1.69 to 3.33 L/min; *p* = 0.52) (Figure S1.3). Significant heterogeneity was evident in the overall treatment effect (*Q* = 35.60, *df* = 6, *I*^2^ = 83%; *p* = 0.00001). Significant subgroup differences and heterogeneity were found in the subgroup analysis, suggesting that BMI had an influence on the treatment effect of this outcome. Publication bias was not assessed as less than ten studies were included in the meta-analysis of this outcome. Sensitivity analysis did not affect the direction or significance of the outcome, showing no difference between IET compared to CET; however, participants with BMI >30 kg/m^2^ had a greater benefit from IET compared to CET.

Four out of 13 studies^4,16,22,26^ assessed VO_2_ at the LAT, demonstrating no significant difference between groups (MD = 0.01 L/min, 95% CI: −0.04 to 0.07 L/min; *p* = 0.65) (Figure S1.4). Meta-analytic results of IET compared to CET reported non-important heterogeneity, suggesting consistency between studies (*Q* = 0.29, *df* = 3, *I*^2^ = 0%; *p* = 0.96), and as a result subgroup analysis were not performed. Publication bias was not assessed as less than ten studies were included in the meta-analysis of this outcome. Sensitivity analysis did not affect the direction or significance of the outcome, showing no difference between IET compared to CET.

### Symptoms

**Figure 3.**
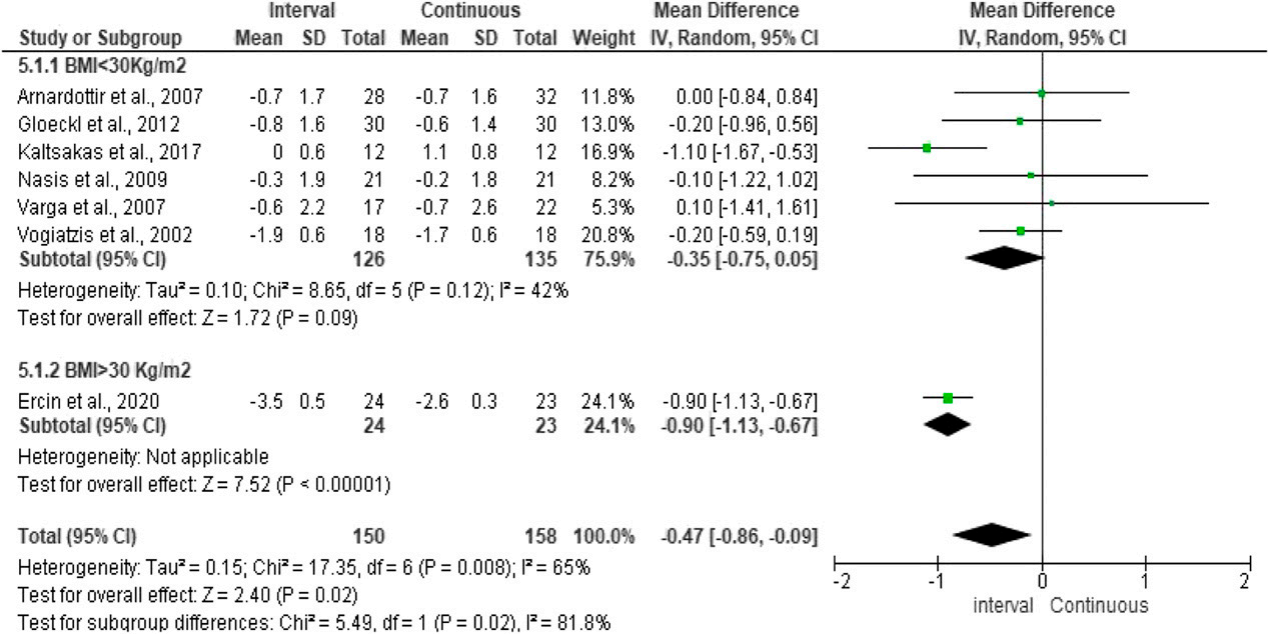
Comparison of the effect of IET versus CET on dyspnoea (Borg’s scale CR 0–10). Subgroup analysis by BMI (<30 kg/m^2^).

Peak dyspnoea was reported in seven studies,^4,15,17,21-23,25^ assessed by the modified Borg CR 0-10 scale.^10^ Pooled results in the primary meta-analysis revealed a significant effect on peak dyspnoea favouring IET compared to CET (MD = −0.47, 95% CI: −0.86 to −0.09; *p* = 0.02) (Figure 3). However, significant heterogeneity was detected in the overall treatment effect for this outcome (*Q* = 17.35, *df* = 6, *I*^2^ = 65%; *p* = 0.08). Significant subgroup differences and heterogeneity were detected, suggesting no difference between IET compared to CET in participants with normal BMI; however, participants with BMI >30 kg/m^2^ had a greater benefit from IET compared to CET. Publication bias was not assessed as less than ten studies were included in the meta-analysis of this outcome. Sensitivity analysis influenced the direction and significance of the outcome showing no differences between the two training modalities.

Peak leg discomfort was reported in five studies^15,17,21-23,25^ assessed by the modified Borg CR 0-10 scale^10^ and in one study^23^ assessed by the Borg RPE scale (6–20). Pooled results in the primary meta-analysis revealed no significant difference between groups on peak leg discomfort (MD = −0.48, 95% Cl: −1.04 to 0.09; *p* = 0.10) (Figure S1.5). Significant heterogeneity was indicated in the overall treatment effect for this outcome (*Q* = 22.01, *df* = 5, *I*^2^ = 77%, *p* = 0.0005). No subgroup differences or heterogeneity were found in the subgroup analysis and presence of publication bias was demonstrated by the asymmetric funnel plot. Sensitivity analysis did not affect the direction of the outcome, showing no differences between the two training modalities.

### Training volume

Calculation of total training volume applied in earlier studies^4,15,16,18-25^ comparing CET and IET modalities, revealed that the total training volume was highly comparable between the two modalities (Figure S4).

## Discussion

This meta-analysis assessed the efficacy of IET versus CET on exercise capacity, cardiorespiratory fitness and exertional symptoms in patients with COPD, CF and LT candidates. To our knowledge, this is the first meta-analysis to investigate the impact of IET compared to CET on physiological responses not only in COPD but also in other CRDs. Furthermore, our review includes five additional RCTs^15,17,20,25,26^ to previous reviews,^5,6^ providing an updated evidence synthesis. Our meta-analysis differs from the review by Sawyer et al.^7^ as it pooled data from RCTs only, therefore providing a review of the highest quality evidence^27^ on the effects of the two training modalities on exercise capacity, cardiorespiratory fitness and exertional symptoms.

The primary findings indicate that IET is superior to CET in improving peak exercise capacity while inducing lower dyspnoea sensations at the limit of peak exercise tolerance. Despite the significantly better overall improvements for the IET on WR_peak_ and dyspnoea, these did not exceed the MID of 4 W for WR_peak_^28^ and 1 unit for dyspnoea points,^29^ respectively. These results including several new studies are in contrast to previous meta-analyses^5,6^ which reported no significant differences between IET and CET for these variables in patients with COPD. Lack of differences were previously attributed to highly comparable total training volume between the two training modalities (Figure S4).^5,6^ However, while total training volume (frequency × duration × length of training programme) may have been matched between IET and CET, improvements in exercise capacity in COPD depend on exercise intensity.^16,30^ Accordingly, despite normalising for total training volume between modalities, higher intensity during IET may have resulted in greater improvements in exercise capacity compared to CET.^8^

Findings for WR_peak_ suggest that IET may yield greater improvements in exercise capacity, compared to CET in patients with CRDs. This may be attributed to greater structural alterations induced by high intensity exercise within the locomotor muscles, thereby enhancing the oxidative potential of these muscles. Earlier work by Morris et al.^13^ suggested that the higher intensity during IET may result in greater improvements in exercise capacity compared to CET. In support of this notion, a recent study^16^ in people with CF demonstrated that IET compared to Cet allowed greater improvements in exercise intensity throughout the training programme, leading to greater improvements in quadriceps muscle strength compared to CET.

It has been proposed that exercise training can partially reverse the shift towards glycolytic fibres in COPD patients.^31^ Quantifiable changes in muscle hypertrophy and fibre-type distribution are noted after high intensity IET, increasing the amount of type-I fibres.^32^ Furthermore, Vogiatzis et al.^33^ supported that IET was more effective than CET in enhancing the expression of anabolic growth hormones [insulin-like growth factor-1 (IGF-1) and myogenic differentiation factor-D (MGF)] that stimulate muscle fibre hypertrophy and protein synthesis.^33^ In 2010, Vogiatzis et al.^34^ reported enhanced muscle hypertrophy after high intensity IET when mRNA expression of both IGF-I and the MGF, an isoform of IGF-I, were significantly higher in both cachectic and non-cachectic COPD patients post-training.^34^ In healthy untrained individuals, high intensity IET promoted the upregulation of muscle growth and mitochondrial pathways.^35^ A previous study^36^ on healthy untrained individuals found that 2 weeks of high intensity IET induced increased protein expression and mitochondrial enzyme activity, leading to enhanced oxidative capacity of the skeletal muscles. Improved tissue oxidative activity of the skeletal muscles during exercise facilitates higher gains in exercise tolerance^36^ and is associated with reduced ventilatory drive to breathe.^37^ The latter may justify the reduced dyspnoea sensations following IET in our meta-analysis.

Four studies^4,21,17,25^ indicated that IET was associated with lower dyspnoea sensations than CET at the limit of tolerance. This is an important finding when considering that IET elicited greater improvements in WR_peak_. A likely explanation for this finding may be that in two of the studies^4,16^ inpatients with COPD showed less ventilatory requirement at an identical submaximal work rate after IET. These adaptations at submaximal levels were associated with clinically meaningful increases in inspiratory capacity (IC), thereby suggesting the mitigation of exercise-induced dynamic hyperinflation^16^ and subsequent dyspnoea sensations. Inspiratory capacity has been found to be a major contributor to endurance capacity, reflecting the operating limits for tidal volume expansion and CO_2_ retention during incremental exercise.^38^ This finding confirms the established relationship between dyspnoea intensity and the degree of dynamic hyperinflation,^39^ where lower dyspnoea during IET might trigger smaller increases in end-expiratory lung volume as compared with CET.^40^

Improvements in the degree of dynamic hyperinflation with interval exercise could be explained by one study^41^ which indicated higher IC values at exercise isotime in COPD patients. Less exercise-induced dynamic hyperinflation was supported by greater tidal volume, inspiratory time and, in turn, lower breathing frequency compared to continuous exercise. Minute ventilation was similar between the two exercise modalities; however, patients could sustain the same level of minute ventilation for prolonged periods of time during interval compared to continuous exercise. In agreement with this study,^41^ evidence by Vogiatzis et al.^42^ demonstrated that comparable levels of minute ventilation between the two exercise modalities were sustained for a threefold amount of time during interval exercise, to the point of exercise limitation. Moreover, Sabapathy et al.^43^ reported significantly lower dynamic hyperinflation during interval exercise in COPD patients, supporting the proposed superiority of this modality in COPD.

The interpretation of the improvements in WR_peak_ and dyspnoea sensations between IET and CET needs to be treated with caution as it may not fully reflect the real effect between the two training modalities. Sensitivity analyses removed four studies^4,16,21,25^ from the primary meta-analysis for WR_peak_ and three studies^4,21,25^ for dyspnoea that exhibited fair quality in the PEDro scale, due to limitations in study designs. The excluded studies failed to report whether allocation sequence was properly concealed, and if any blinding of subjects, therapists or assessors was conducted. Therefore, elimination of the potential risk of bias and the production of spurious summary measures that overestimate the treatment effects should be achieved. Pooled results of sensitivity analyses for both outcomes revealed no significant differences between IET and CET, alternating the direction and the significance (*p* = 0.93) of the overall effect estimates from the primary meta-analyses.

Subgroup analysis was only feasible for dyspnoea, where significant heterogeneity was presented suggesting variation across studies. It is likely that the variability in the intervention effects is a result of clinical diversity among subjects across the different studies. Indeed, this hypothesis is further supported by the fact that there was no identification of tailoring exercise protocols on patients’ specific characteristics or comorbidities. Precise exercise tailoring to patients’ needs and capabilities greatly affects the physiological responses as different exercise prescriptions contribute to different physiological responses. Consequently, to explore heterogeneity, we conducted subgroup analysis on participants with BMI <30 kg/m^2^ as indicated in the methods section (when *I*^2^ >40%). Hence, subgroup analysis for dyspnoea demonstrated significant differences (*p* = 0.02) between the two subsets of studies, suggesting that IET was superior to CET for obese participants with COPD; however, no differences were demonstrated between the training modalities for the subset of studies with participants with a BMI < 30 kg/m^2^.

The presence of obesity constitutes an important comorbid factor associated with increased dyspnoea, elevated work of breathing and decreased exercise capacity than normal individuals, independently of the presence of airflow limitation.^44^ Our results demonstrated that higher BMI (>30 kg/m^2^) could influence the impact of the training modality, suggesting that IET could be more beneficial than CET for obese participants with COPD, alleviating dyspnoea sensations to a greater extent and increasing exercise capacity during cycling. It is well documented that high intensity aerobic exercise training is highly beneficial for the components of metabolic syndrome in obese individuals.^45^ This fact together with the obesity ‘paradox’, where individuals with COPD and obesity develop less dynamic hyperinflation during cycling than normal weight, suggests that IET may be an effective training modality to improve exercise capacity and dyspnoea during cycling in these patients.^45^ Hence, for a successful pulmonary rehabilitation delivery, it is necessary to sufficiently address comorbidities such as obesity, by tailoring carefully exercise recommendations to patients’ needs and capabilities.

When observing cardiovascular and metabolic responses, our results are consistent with Beauchamp et al.^5^ and Zainuldin et al.^6^ who reported no differences between the two training modalities. Sensitivity analysis revealed no change in the direction of the overall effect from the primary meta-analysis for VO_2__peak_, HR_peak_, VE_peak_, LAT and leg discomfort. When investigating heterogeneity for several of the outcomes (HR_peak_, VE_peak_ and leg discomfort), there were significant subgroup differences (*p* = 0.00001 for HR_peak_ and *p* = 0.01 for VE_peak_) demonstrating that higher BMI (>30 kg/m^2^) could influence the impact of the training modality, thereby suggesting that IET could be more beneficial than CET in terms of minute ventilation for obese participants with COPD.

### Study limitations and Implications

Limitations involved the small sample sizes of included studies and the predominance of COPD patients. There is a lack of RCTs investigating the impact of IET compared to CET on exercise capacity, symptoms and HRQoL on respiratory conditions other than COPD. Given that IET is associated with reduced symptoms and exercise-induced arterial oxygen desaturation, future larger scale and high-quality studies investigating the effectiveness of this modality in comparison to CET in patients with other types of respiratory disease than COPD are warranted. Furthermore, some of the trials had fair methodological quality when assessed by the PEDro scale. Since the overall study quality was fair to good, caution is needed when interpreting the results. Many of the included RCTs were unable to blind patients or therapists to the treatment group, leaving the results exposed to bias. Another limitation was the decision to include only English language articles; however, this was necessary as access to a translator was not available. Evidence^46^ supports that the exclusion of non-English articles affects only 5% of the estimates of effect. Funnel plots were not feasible due to insufficient number of studies for most of the physiological responses, apart from WR_peak_. Locating and including unpublished studies and unpublished outcomes of published studies would be a recommendation for an updated version of this meta-analysis to provide a better estimate of effectiveness. Additionally, the authors acknowledge that the registration of this study in PROSPERO (prospective register of systematic reviews) was conducted after the formal screening of search results against eligibility criteria. Since we appreciate the importance of trial registration, we believe that this would be a crucial mechanism for eliminating the impact of publication bias in future meta-analysis. Furthermore, since different interval protocols may give different results as previously noted by Morris et al.^13^ more studies should address these training parameters and their impact on physiological adaptations. Future studies should investigate the optimal total training volume for producing the most beneficial training adaptations specifically tailored to patients’ needs. Finally, our meta-analysis was focused on data obtained from cardiopulmonary exercise testing on the cycle ergometer but not on field-based walking tests.

## Conclusions

This meta-analysis indicates that IET is superior to CET in patients with CRDs, in improving peak exercise capacity and lessening breathlessness at the limit of tolerance during exercise. Physiological adaptations after IET would be beneficial for the performance of daily activities with lower breathlessness for longer periods of time. Thus, interval exercise may be a preferable training option in respiratory patients unable to sustain continuous exercise due to profound breathlessness and exercise-induced arterial hypoxaemia.

## Supplemental Material

sj-pdf-1-crd-10.1177_14799731211041506 – Supplemental Material for Effect of interval compared to continuous exercise training on physiological responses in patients with chronic respiratory diseases: A systematic review and meta-analysis Click here for additional data file. Supplemental Material, sj-pdf-1-crd-10.1177_14799731211041506 for Effect of interval compared to continuous exercise training on physiological responses in patients with chronic respiratory diseases: A systematic review and meta-analysis by Charikleia Alexiou, Lesley Ward, Emily Hume, Matthew Armstrong, Mick Wilkinson and Ioannis Vogiatzis in Chronic Respiratory Disease
