# Supplementary material for: Effect of interval compared to continuous exercise training on physiological responses in patients with chronic respiratory diseases: A systematic review and meta-analysis
Source: Chron Respir Dis. 2021 Oct 19;18:14799731211041506. doi: 10.1177/14799731211041506 (PMC8532221; doi:10.1177/14799731211041506)

Supplementary Material

Methods

Search strategy and screening

Table S1. PubMed Search Strategy

Search	Query
**#1**	**(("lung diseases"[MeSH Terms] OR "chronic lung disease"[Text Word]) OR "chronic respiratory disease"[Text Word]) OR "cystic fibrosis"[Text Word]) OR "pulmonary arterial hypertension"[Text Word]) OR "pulmonary hypertension"[Text Word]) OR "ILD"[Text Word]) OR "pulmonary fibrosis"[Text Word]) OR "asthma"[Text Word]) OR "chronic obstructive pulmonary disease"[Text Word]) OR "COPD"[Text Word]) OR "lung transplantation"[MeSH Terms]) OR "bronch*"[Text Word]) OR "pulmonary disease"[Text Word]))**
**#2**	**(("interval"[Text Word] OR "intermittent"[Text Word]) OR "high intensity"[Text Word])**
**#3**	**(("exercise"[MeSH Terms] OR "exercise therapy"[MeSH Terms]) OR "rehabilitation"[MeSH Terms]) OR "rehabilit*"[Text Word]) OR "exercis*"[Text Word]) OR "train*"[Text Word]) OR "continuous"[Text Word]) OR "endurance"[Text Word]) OR "aerobic"[Text Word]) OR "moderate intensity"[Text Word]))**
**#4**	**(("exercise tolerance"[MeSH Terms] OR "exercise tolerance"[Text Word]) OR "exercise capacity"[Text Word]) OR "quality of life"[MeSH Terms]) OR "quality of life"[Text Word]) OR "oxygen consumption"[MeSH Terms]) OR "treatment outcome"[MeSH Terms]) OR "dyspnea"[MeSH Terms]) OR "dyspnea"[Text Word]) OR "physical fitness"[MeSH Terms]) OR "forced expiratory volume"[MeSH Terms]) OR "leg fatigue"[Text Word]) OR "leg discomfort"[Text Word]) OR "breathlessness"[Text Word])**
**#5**	**1 AND 2 AND 3 AND 4**

Results

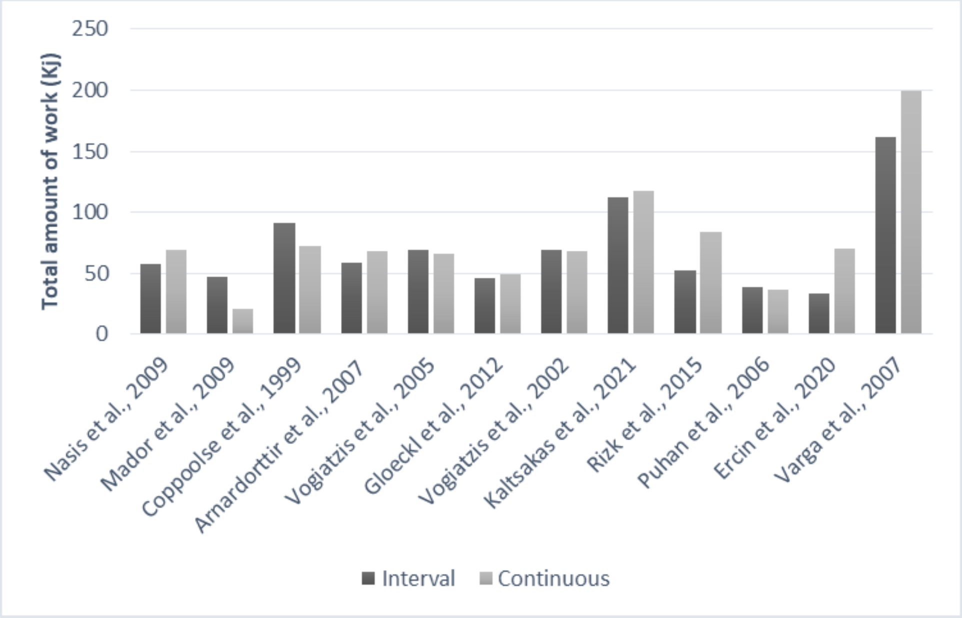

Figure S4. Comparison of the total volume of training between IET and CET for the duration of each programme as per study design.

Analysis S1. Meta-analysis and subgroup analysis

Figure S1.1. Comparison of the effect of IET versus CET on peak oxygen uptake ($VO2_{peak}$) in L/min.

	Interval			Continuous				Mean Difference
Study or Subgroup	Mean	SD	Total	Mean	SD	Total	Weight	IV, Random, 95% CI
Arnardottir et al., 2007	0.1	0.2	25	0.1	0.2	28	10.1%	0.00 [-0.11, 0.11]
Brostand et al., 2013	0.1	0.4	10	0.1	0.04	7	1.9%	0.00 [-0.25, 0.25]
Coppoolse et al., 1999	0.09	0.18	10	0.15	0.24	11	3.6%	-0.06 [-0.24, 0.12]
Ercin et al., 2020	0.64	0.002	24	0.58	0.3	23	7.8%	0.06 [-0.06, 0.18]
Kaltsakas et al., 2017	0.1	0.06	12	0.1	0.06	12	50.9%	0.00 [-0.05, 0.05]
Mador et al., 2009	0.1	0.4	21	0.2	0.5	20	1.5%	-0.10 [-0.38, 0.18]
Varga et al., 2007	0.08	0.2	17	0.1	0.2	22	7.3%	-0.02 [-0.15, 0.11]
Vogiatzis et al., 2002	0.1	0.2	18	0	0.1	18	11.0%	0.10 [-0.00, 0.20]
Vogiatzis et al., 2005	0.1	0.2	10	0.1	0.1	9	6.0%	0.00 [-0.14, 0.14]
**Total (95% CI)**			**147**			**150**	**100.0%**	**0.01 [-0.02, 0.04]**

Heterogeneity: Tau² = 0.00; Chi² = 5.17, df = 8 (P = 0.74); I² = 0%
Test for overall effect: Z = 0.60 (P = 0.55)

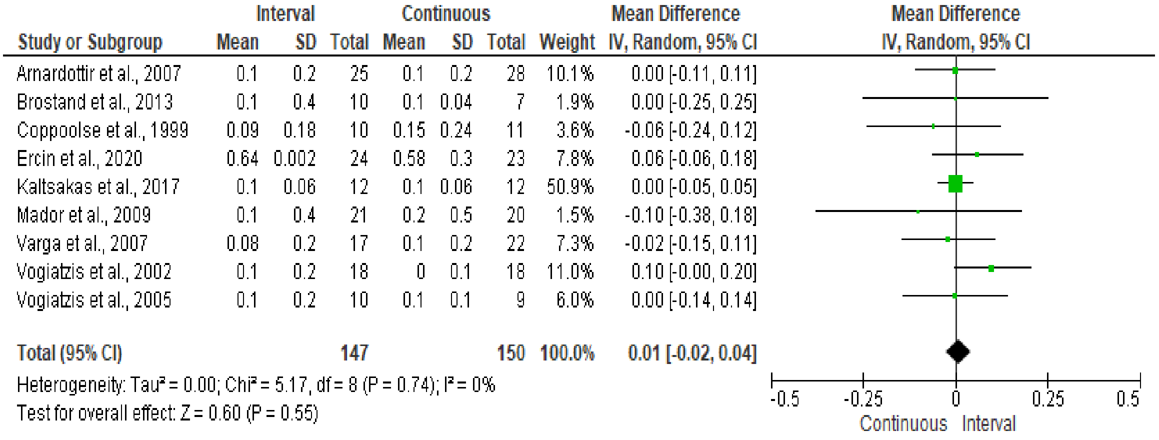

Figure S1.2. Comparison of the effect of IET versus CET on peak heart rate (HR_{peak}) in beats/min. Subgroup analysis by BMI (<30Kg/m^2).

Study or Subgroup	Interval Mean	Interval SD	Interval Total	Continuous Mean	Continuous SD	Continuous Total	Weight	Mean Difference IV, Random, 95% CI
**7.1.1 BMI<30Kg/m2**								
Arnardottir et al., 2007	1	18	28	-1	2	32	13.0%	2.00 [-4.70, 8.70]
Brostand et al., 2013	-4	2	10	-1	4	7	18.4%	-3.00 [-6.21, 0.21]
Kaltsakas et al., 2017	7	1	12	5	1	12	20.8%	2.00 [1.20, 2.80]
Mador et al., 2009	0	13	21	-4	18	20	9.2%	4.00 [-5.65, 13.65]
Varga et al., 2007	-1	19	17	-5	4	22	9.7%	4.00 [-5.19, 13.19]
Vogiatzis et al., 2002	13	16.8	18	10	16.8	18	7.9%	3.00 [-7.98, 13.98]
**Subtotal (95% CI)**			**106**			**111**	**79.1%**	**0.97 [-1.65, 3.59]**
Heterogeneity: Tau² = 3.96; Chi² = 9.27, df = 5 (P = 0.10); I² = 46%								
Test for overall effect: Z = 0.73 (P = 0.47)								
**7.1.2 BMI>30Kg/m2**								
Ercin et al., 2020	-11	1.2	24	-6.5	0.9	23	20.9%	-4.50 [-5.10, -3.90]
**Subtotal (95% CI)**			**24**			**23**	**20.9%**	**-4.50 [-5.10, -3.90]**
Heterogeneity: Not applicable								
Test for overall effect: Z = 14.58 (P < 0.00001)								
**Total (95% CI)**			**130**			**134**	**100.0%**	**0.18 [-3.73, 4.09]**
Heterogeneity: Tau² = 18.95; Chi² = 167.15, df = 6 (P < 0.00001); I² = 96%								
Test for overall effect: Z = 0.09 (P = 0.93)								
Test for subgroup differences: Chi² = 15.89, df = 1 (P < 0.0001), I² = 93.7%								

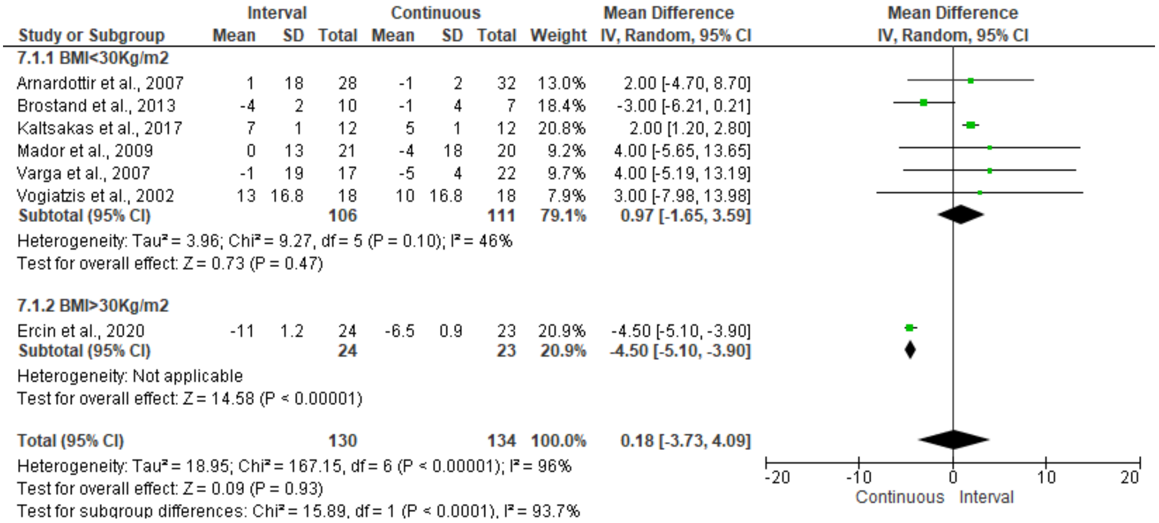

Figure S1.3. Comparison of the effect of IET versus CET on peak minute ventilation (VE_{peak}) in L/min. Subgroup analysis by BMI (<30Kg/m^2).

Study or Subgroup	Interval Mean	Interval SD	Interval Total	Continuous Mean	Continuous SD	Continuous Total	Weight	Mean Difference IV, Random, 95% CI
**4.1.1 BMI<30Kg/m2**								
Arnardottir et al., 2007	1.7	5.5	25	3.2	6.6	28	16.5%	-1.50 [-4.76, 1.76]
Brostand et al., 2013	3	4	10	0	14	7	4.5%	3.00 [-7.66, 13.66]
Kaltsakas et al., 2017	5.2	4.2	12	10.1	4.5	12	15.8%	-4.90 [-8.38, -1.42]
Mador et al., 2009	4.3	14.5	21	3.7	19.2	20	4.6%	0.60 [-9.85, 11.05]
Varga et al., 2007	3	3	17	0	1	22	21.1%	3.00 [1.51, 4.49]
Vogiatzis et al., 2002	3.3	6.4	18	2.5	4.6	18	15.4%	0.80 [-2.84, 4.44]
**Subtotal (95% CI)**			**103**			**107**	**77.8%**	**-0.15 [-3.31, 3.02]**
Heterogeneity: Tau² = 9.68; Chi² = 20.22, df = 5 (P = 0.001); I² = 75%								
Test for overall effect: Z = 0.09 (P = 0.93)								
**4.1.2 BMI>30 Kg/m2**								
Ercin et al., 2020	21.5	1.6	24	17.3	1.5	23	22.2%	4.20 [3.31, 5.09]
**Subtotal (95% CI)**			**24**			**23**	**22.2%**	**4.20 [3.31, 5.09]**
Heterogeneity: Not applicable								
Test for overall effect: Z = 9.29 (P < 0.00001)								
**Total (95% CI)**			**127**			**130**	**100.0%**	**0.82 [-1.69, 3.33]**
Heterogeneity: Tau² = 7.20; Chi² = 35.60, df = 6 (P < 0.00001); I² = 83%								
Test for overall effect: Z = 0.64 (P = 0.52)								
Test for subgroup differences: Chi² = 6.72, df = 1 (P = 0.010), I² = 85.1%								

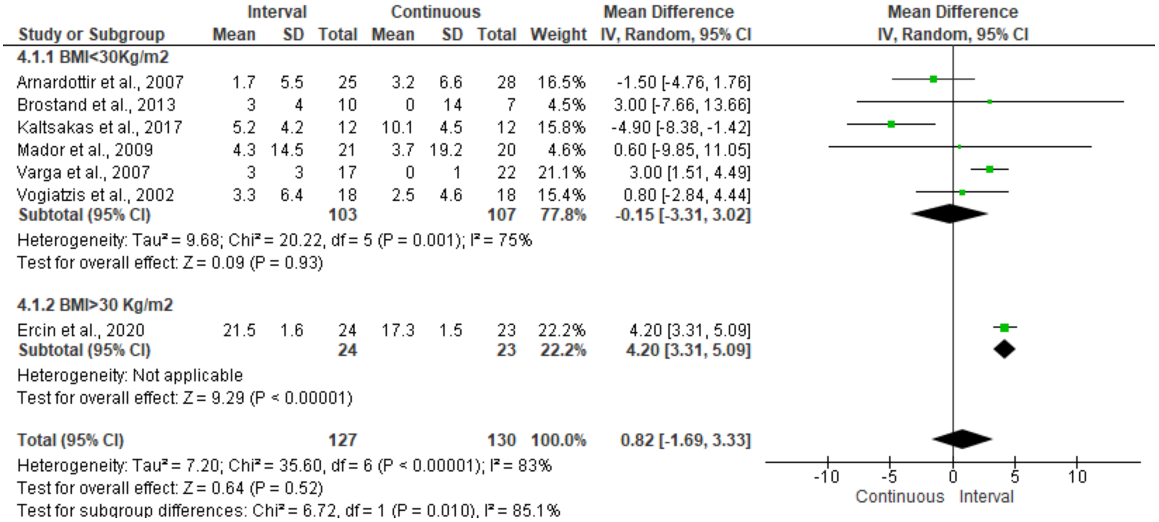

Figure S1.4. Comparison of the effect of IET versus CET on lactate threshold (LAT) in L/min. Subgroup analysis by BMI (<30Kg/m2).

Study or Subgroup	Interval Mean	Interval SD	Interval Total	Continuous Mean	Continuous SD	Continuous Total	Weight	Mean Difference IV, Fixed, 95% CI
Brostand et al., 2013	0.1	0.13	10	0.07	0.03	7	41.3%	0.03 [-0.05, 0.11]
Varga et al., 2007	0.1	0.2	17	0.1	0.1	22	26.7%	0.00 [-0.10, 0.10]
Vogiatzis et al., 2002	0.1	0.2	19	0.1	0.2	18	17.3%	0.00 [-0.13, 0.13]
Vogiatzis et al., 2005	0.1	0.2	10	0.1	0.1	9	14.7%	0.00 [-0.14, 0.14]
**Total (95% CI)**			**56**			**56**	**100.0%**	**0.01 [-0.04, 0.07]**
Heterogeneity: Chi² = 0.29, df = 3 (P = 0.96); I² = 0%								
Test for overall effect: Z = 0.45 (P = 0.65)								

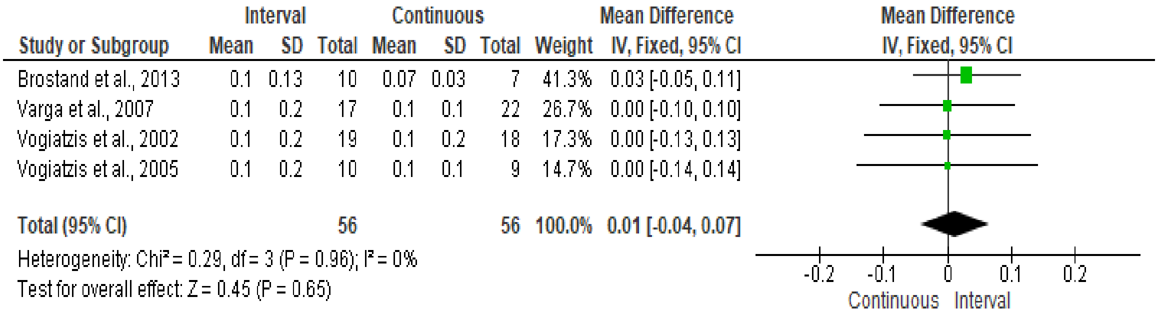

Figure S1.5. Comparison of the effect of IET versus CET on leg discomfort (Borg's scale CR 0-10). Subgroup analysis by BMI (<30Kg/m2).

Study or Subgroup	Interval Mean	Interval SD	Interval Total	Continuous Mean	Continuous SD	Continuous Total	Weight	Mean Difference IV, Random, 95% CI
**6.1.1 BMI<30Kg/m2**								
Arnardottir et al., 2007	-0.3	1.4	28	-0.6	1.3	28	18.8%	0.30 [-0.41, 1.01]
Gloeckl et al., 2012	-0.9	1.9	30	-0.7	2.1	30	14.4%	-0.20 [-1.21, 0.81]
Kaltsakas et al., 2017	-1.3	0.1	12	0	0.6	12	24.3%	-1.30 [-1.64, -0.96]
Nasis et al., 2009	0.3	1.4	21	-0.2	3	21	10.0%	0.50 [-0.92, 1.92]
Varga et al., 2007	-0.8	2.4	17	-0.3	3	22	7.8%	-0.50 [-2.20, 1.20]
**Subtotal (95% CI)**			**108**			**113**	**75.3%**	**-0.30 [-1.20, 0.60]**
Heterogeneity: Tau² = 0.78; Chi² = 21.95, df = 4 (P = 0.0002); I² = 82%								
Test for overall effect: Z = 0.65 (P = 0.52)								
**6.1.2 BMI>30Kg/m2**								
Ercin et al., 2020	-3	0.5	24	-2.2	0.6	23	24.7%	-0.80 [-1.12, -0.48]
**Subtotal (95% CI)**			**24**			**23**	**24.7%**	**-0.80 [-1.12, -0.48]**
Heterogeneity: Not applicable								
Test for overall effect: Z = 4.95 (P < 0.00001)								
**Total (95% CI)**			**132**			**136**	**100.0%**	**-0.48 [-1.04, 0.09]**
Heterogeneity: Tau² = 0.31; Chi² = 22.01, df = 5 (P = 0.0005); I² = 77%								
Test for overall effect: Z = 1.65 (P = 0.10)								
Test for subgroup differences: Chi² = 1.05, df = 1 (P = 0.30), I² = 5.2%								

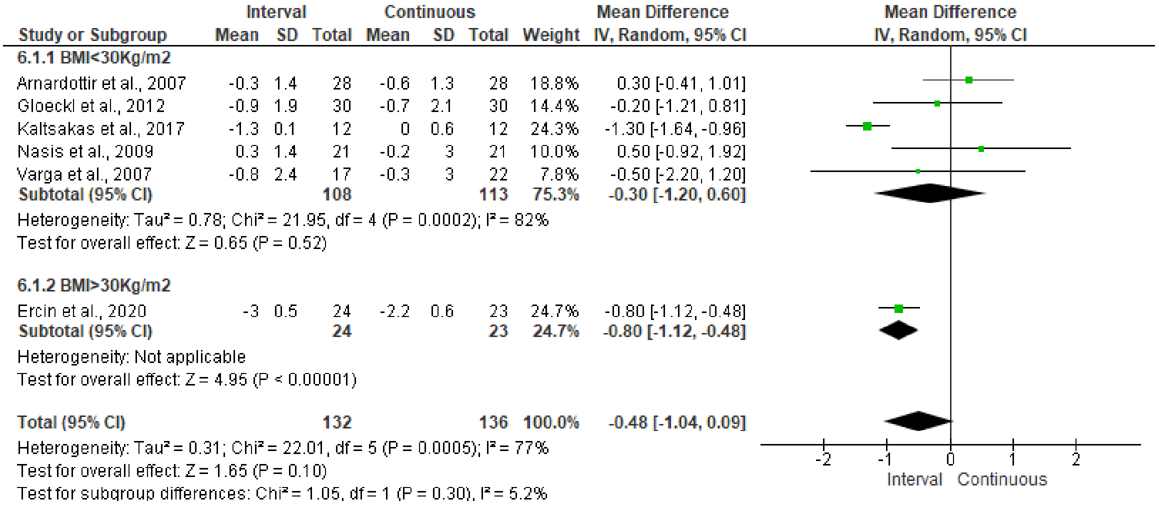

Analysis S2. Sensitivity Analysis

Figure S2.1. Forest plot of comparison between good to excellent quality studies and poor to fair quality studies rated on PEDro scale on peak work rate (WR_{peak}).

Study or Subgroup	Interval Mean	Interval SD	Interval Total	Continuous Mean	Continuous SD	Continuous Total	Weight	Mean Difference IV, Random, 95% CI
**1.1.1 Good to excellent quality on PEDro scale**								
Arnardottir et al., 2007	11	7	28	11	12	32	10.3%	0.00 [-4.90, 4.90]
Coppoolse et al., 1999	12	17	10	5	18	11	1.1%	7.00 [-7.97, 21.97]
Gloeckl et al., 2012	14	7	30	13	6	30	22.6%	1.00 [-2.30, 4.30]
Kaltsakas et al., 2017	19	4	12	16	10	12	6.6%	3.00 [-3.09, 9.09]
Mador et al., 2009	10	13	21	11.5	13.1	20	3.9%	-1.50 [-9.49, 6.49]
Puhan et al., 2006	8.5	12	43	8.7	10.4	44	11.1%	-0.20 [-4.92, 4.52]
Varga et al., 2007	14	12	17	12	9	22	5.3%	2.00 [-4.83, 8.83]
**Subtotal (95% CI)**			**161**			**171**	**60.8%**	**0.87 [-1.14, 2.88]**
Heterogeneity: Tau² = 0.00; Chi² = 1.88, df = 6 (P = 0.93); I² = 0%								
Test for overall effect: Z = 0.85 (P = 0.40)								
**1.1.2 Poor to fair quality on PEDro scale**								
Ercin et al., 2020	52	7	24	46.7	2	23	29.0%	5.30 [2.38, 8.22]
Nasis et al., 2009	14	9.2	21	10	9.2	21	8.0%	4.00 [-1.56, 9.56]
Vogiatzis et al., 2002	14	15.2	18	13	23.5	18	1.5%	1.00 [-11.93, 13.93]
Vogiatzis et al., 2005	10	15.2	10	9	23.5	9	0.8%	1.00 [-17.01, 19.01]
**Subtotal (95% CI)**			**73**			**71**	**39.2%**	**4.79 [2.28, 7.30]**
Heterogeneity: Tau² = 0.00; Chi² = 0.69, df = 3 (P = 0.87); I² = 0%								
Test for overall effect: Z = 3.74 (P = 0.0002)								
**Total (95% CI)**			**234**			**242**	**100.0%**	**2.40 [0.83, 3.97]**
Heterogeneity: Tau² = 0.00; Chi² = 8.29, df = 10 (P = 0.60); I² = 0%								
Test for overall effect: Z = 3.00 (P = 0.003)								
Test for subgroup differences: Chi² = 5.71, df = 1 (P = 0.02), I² = 82.5%								

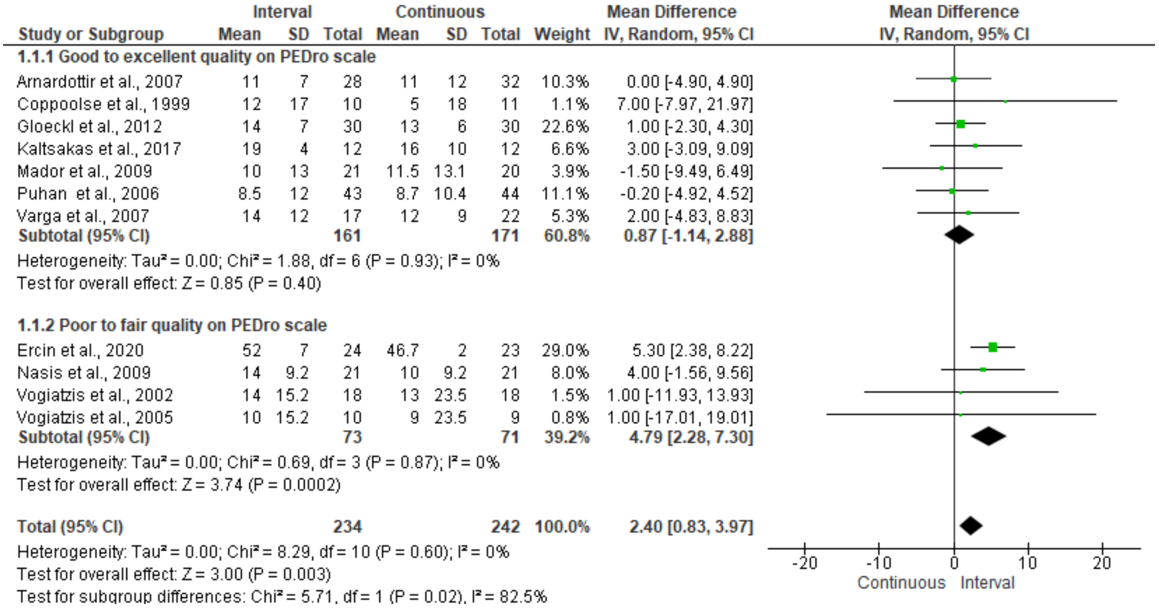

Figure S2.2. Forest plot of comparison between good to excellent quality studies and poor to fair quality studies rated on PEDro scale on peak oxygen uptake (VO2peak).

Study or Subgroup	Interval Mean	Interval SD	Interval Total	Continuous Mean	Continuous SD	Continuous Total	Weight	Mean Difference IV, Random, 95% CI
**2.1.1 Good to excellent quality on PEDro scale**								
Arnardottir et al., 2007	0.1	0.2	25	0.1	0.2	28	10.1%	0.00 [-0.11, 0.11]
Brostand et al., 2013	0.1	0.4	10	0.1	0.04	7	1.9%	0.00 [-0.25, 0.25]
Coppoolse et al., 1999	0.09	0.18	10	0.15	0.24	11	3.6%	-0.06 [-0.24, 0.12]
Kaltsakas et al., 2017	0.1	0.06	12	0.1	0.06	12	50.9%	0.00 [-0.05, 0.05]
Mador et al., 2009	0.1	0.4	21	0.2	0.5	20	1.5%	-0.10 [-0.38, 0.18]
Varga et al., 2007	0.08	0.2	17	0.1	0.2	22	7.3%	-0.02 [-0.15, 0.11]
**Subtotal (95% CI)**			**95**			**100**	**75.2%**	**-0.01 [-0.05, 0.03]**
Heterogeneity: Tau² = 0.00; Chi² = 0.90, df = 5 (P = 0.97); I² = 0%								
Test for overall effect: Z = 0.34 (P = 0.73)								
**2.1.2 Poor to fair quality on PEDro scale**								
Ercin et al., 2020	0.64	0.002	24	0.58	0.3	23	7.8%	0.06 [-0.06, 0.18]
Vogiatzis et al., 2002	0.1	0.2	18	0	0.1	18	11.0%	0.10 [-0.00, 0.20]
Vogiatzis et al., 2005	0.1	0.2	10	0.1	0.1	9	6.0%	0.00 [-0.14, 0.14]
**Subtotal (95% CI)**			**52**			**50**	**24.8%**	**0.06 [-0.01, 0.13]**
Heterogeneity: Tau² = 0.00; Chi² = 1.27, df = 2 (P = 0.53); I² = 0%								
Test for overall effect: Z = 1.80 (P = 0.07)								
**Total (95% CI)**			**147**			**150**	**100.0%**	**0.01 [-0.02, 0.04]**
Heterogeneity: Tau² = 0.00; Chi² = 5.17, df = 8 (P = 0.74); I² = 0%								
Test for overall effect: Z = 0.60 (P = 0.55)								
Test for subgroup differences: Chi² = 3.00, df = 1 (P = 0.08), I² = 66.7%								

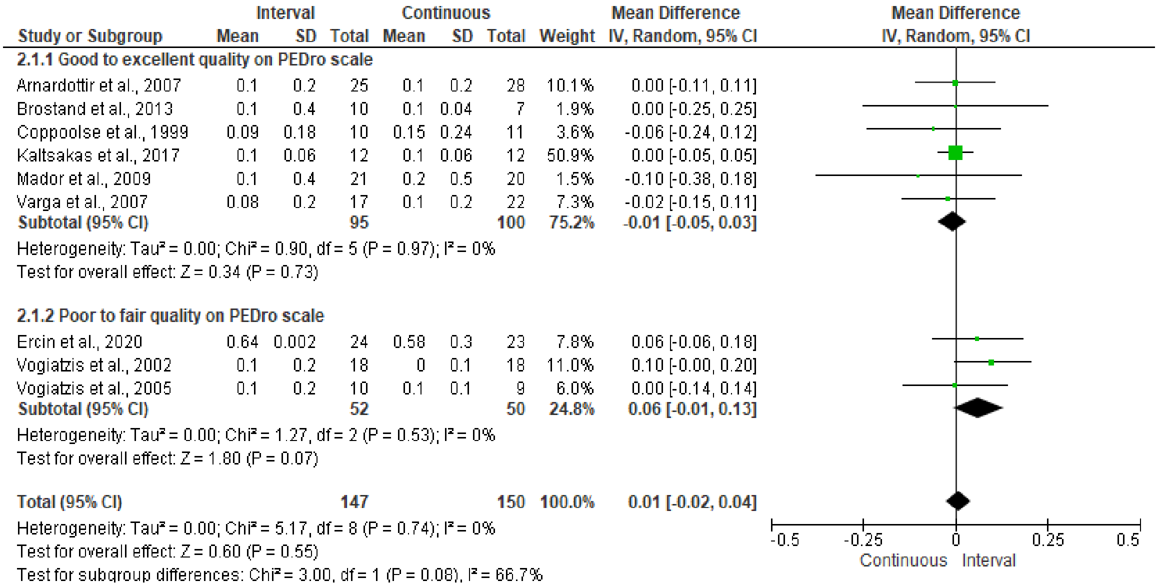

Figure S2.3. Forest plot of comparison between good to excellent quality studies and poor to fair quality studies rated on PEDro scale on peak heart rate (HR_{peak}).

	Interval			Continuous				Mean Difference
**Study or Subgroup**	**Mean**	**SD**	**Total**	**Mean**	**SD**	**Total**	**Weight**	**IV, Random, 95% CI**
**7.1.1 Good to excellent quality on PEDro scaleNew Subgroup**								
Arnardottir et al., 2007	1	18	28	-1	2	32	13.0%	2.00 [-4.70, 8.70]
Brostand et al., 2013	-4	2	10	-1	4	7	18.4%	-3.00 [-6.21, 0.21]
Kaltsakas et al., 2017	7	1	12	5	1	12	20.8%	2.00 [1.20, 2.80]
Mador et al., 2009	0	13	21	-4	18	20	9.2%	4.00 [-5.65, 13.65]
Varga et al., 2007	-1	19	17	-5	4	22	9.7%	4.00 [-5.19, 13.19]
**Subtotal (95% CI)**			**88**			**93**	**71.2%**	**0.89 [-2.04, 3.81]**
Heterogeneity: $Tau^2 = 5.11$; $Chi^2 = 9.22$, df = 4 (P = 0.06); $I^2 = 57\%$								
Test for overall effect: Z = 0.59 (P = 0.55)								
**7.1.2 Poor to fair quality on PEDro scale**								
Ercin et al., 2020	-11	1.2	24	-6.5	0.9	23	20.9%	-4.50 [-5.10, -3.90]
Vogiatzis et al., 2002	13	16.8	18	10	16.8	18	7.9%	3.00 [-7.98, 13.98]
**Subtotal (95% CI)**			**42**			**41**	**28.8%**	**-2.83 [-8.94, 3.28]**
Heterogeneity: $Tau^2 = 12.40$; $Chi^2 = 1.79$, df = 1 (P = 0.18); $I^2 = 44\%$								
Test for overall effect: Z = 0.91 (P = 0.36)								
**Total (95% CI)**			**130**			**134**	**100.0%**	**0.18 [-3.73, 4.09]**
Heterogeneity: $Tau^2 = 18.95$; $Chi^2 = 167.15$, df = 6 (P < 0.00001); $I^2 = 96\%$								
Test for overall effect: Z = 0.09 (P = 0.93)								
Test for subgroup differences: $Chi^2 = 1.16$, df = 1 (P = 0.28), $I^2 = 13.7\%$								

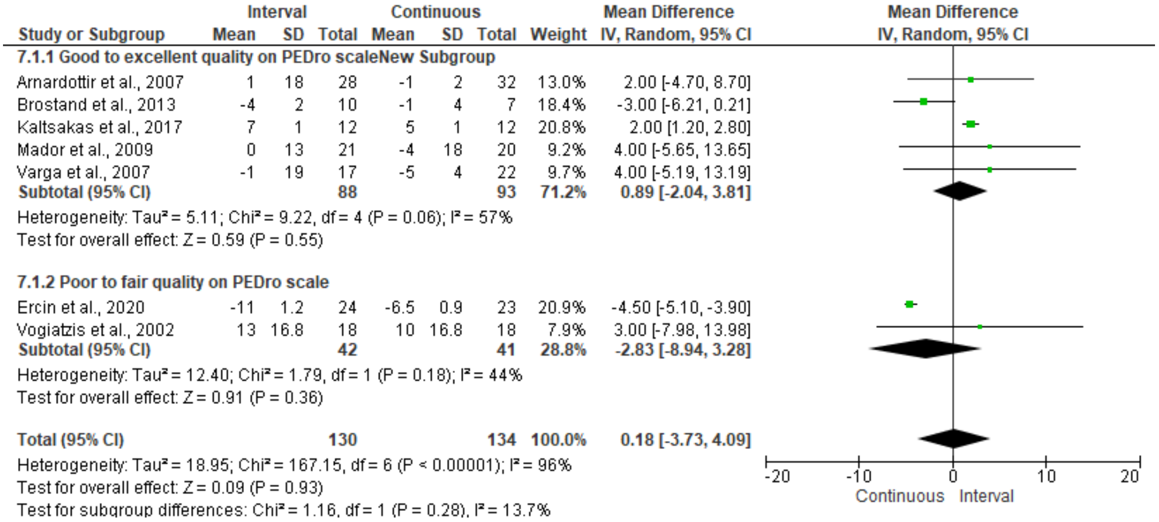

Figure S2.4. Forest plot of comparison between good to excellent quality studies and poor to fair quality studies rated on PEDro scale on peak minute ventilation (VE_{peak}).

	Interval			Continuous				Mean Difference
**Study or Subgroup**	**Mean**	**SD**	**Total**	**Mean**	**SD**	**Total**	**Weight**	**IV, Random, 95% CI**
**4.1.1 Good to excellent quality on PEDro scaleNew Subgroup**								
Arnardottir et al., 2007	1.7	5.5	25	3.2	6.6	28	16.5%	-1.50 [-4.76, 1.76]
Brostand et al., 2013	3	4	10	0	14	7	4.5%	3.00 [-7.66, 13.66]
Kaltsakas et al., 2017	5.2	4.2	12	10.1	4.5	12	15.8%	-4.90 [-8.38, -1.42]
Mador et al., 2009	4.3	14.5	21	3.7	19.2	20	4.6%	0.60 [-9.85, 11.05]
Varga et al., 2007	3	3	17	0	1	22	21.1%	3.00 [1.51, 4.49]
**Subtotal (95% CI)**			**85**			**89**	**62.4%**	**-0.37 [-4.40, 3.66]**
Heterogeneity: $Tau^2 = 13.73$; $Chi^2 = 20.15$, df = 4 (P = 0.0005); $I^2 = 80\%$								
Test for overall effect: Z = 0.18 (P = 0.86)								
**4.1.2 Poor to fair quality on PEDro scale**								
Ercin et al., 2020	21.5	1.6	24	17.3	1.5	23	22.2%	4.20 [3.31, 5.09]
Vogiatzis et al., 2002	3.3	6.4	18	2.5	4.6	18	15.4%	0.80 [-2.84, 4.44]
**Subtotal (95% CI)**			**42**			**41**	**37.6%**	**2.98 [-0.22, 6.18]**
Heterogeneity: $Tau^2 = 3.95$; $Chi^2 = 3.16$, df = 1 (P = 0.08); $I^2 = 68\%$								
Test for overall effect: Z = 1.82 (P = 0.07)								
**Total (95% CI)**			**127**			**130**	**100.0%**	**0.82 [-1.69, 3.33]**
Heterogeneity: $Tau^2 = 7.20$; $Chi^2 = 35.60$, df = 6 (P < 0.00001); $I^2 = 83\%$								
Test for overall effect: Z = 0.64 (P = 0.52)								
Test for subgroup differences: $Chi^2 = 1.63$, df = 1 (P = 0.20), $I^2 = 38.6\%$								

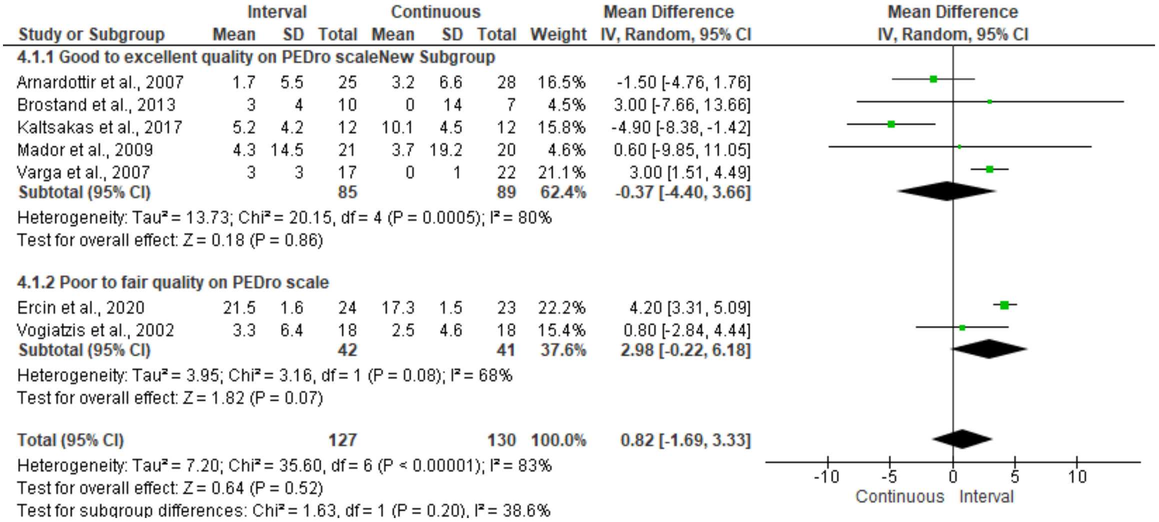

Figure S2.5. Forest plot of comparison between good to excellent quality studies and poor to fair quality studies rated on PEDro scale on lactate threshold (LAT).

Study or Subgroup	Interval Mean	Interval SD	Interval Total	Continuous Mean	Continuous SD	Continuous Total	Weight	Mean Difference IV, Fixed, 95% CI
**3.1.1 Good to excellent quality on PEDro scale**								
Brostand et al., 2013	0.1	0.13	10	0.07	0.03	7	41.3%	0.03 [-0.05, 0.11]
Varga et al., 2007	0.1	0.2	17	0.1	0.1	22	26.7%	0.00 [-0.10, 0.10]
**Subtotal (95% CI)**			**27**			**29**	**68.0%**	**0.02 [-0.05, 0.08]**
Heterogeneity: Chi² = 0.19, df = 1 (P = 0.66); I² = 0%								
Test for overall effect: Z = 0.55 (P = 0.58)								
**3.1.2 Poor to fair quality on PEDro scale**								
Vogiatzis et al., 2002	0.1	0.2	19	0.1	0.2	18	17.3%	0.00 [-0.13, 0.13]
Vogiatzis et al., 2005	0.1	0.2	10	0.1	0.1	9	14.7%	0.00 [-0.14, 0.14]
**Subtotal (95% CI)**			**29**			**27**	**32.0%**	**0.00 [-0.09, 0.09]**
Heterogeneity: Chi² = 0.00, df = 1 (P = 1.00); I² = 0%								
Test for overall effect: Z = 0.00 (P = 1.00)								
**Total (95% CI)**			**56**			**56**	**100.0%**	**0.01 [-0.04, 0.07]**
Heterogeneity: Chi² = 0.29, df = 3 (P = 0.96); I² = 0%								
Test for overall effect: Z = 0.45 (P = 0.65)								
Test for subgroup differences: Chi² = 0.10, df = 1 (P = 0.76), I² = 0%								

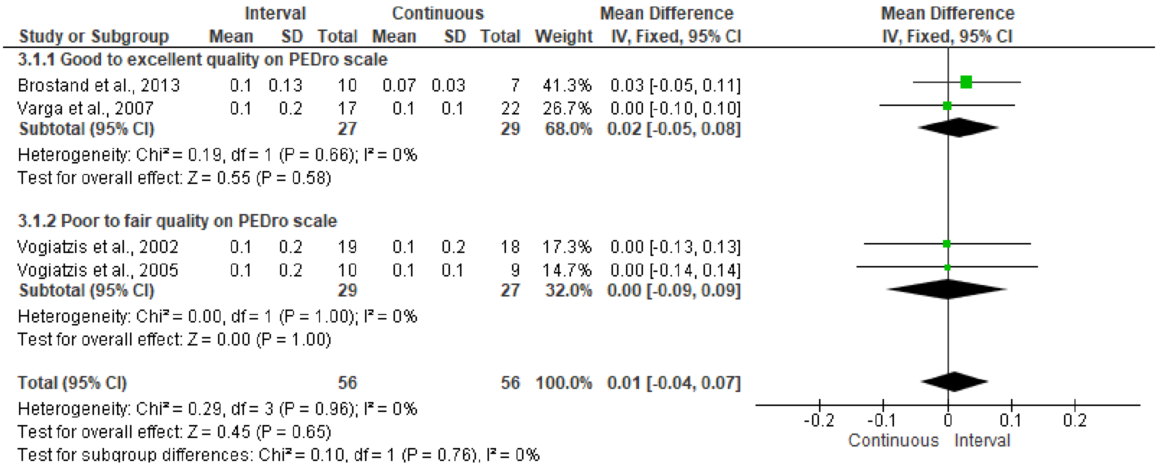

Figure S2.6. Forest plot of comparison between good to excellent quality studies and poor to fair quality studies rated on PEDro scale on dyspnoea (Borg's scale CR 0-10).

Study or Subgroup	Interval Mean	Interval SD	Interval Total	Continuous Mean	Continuous SD	Continuous Total	Weight	Mean Difference IV, Random, 95% CI
**5.1.1 Good to excellent quality on PEDro scale**								
Arnardottir et al., 2007	-0.7	1.7	28	-0.7	1.6	32	11.8%	0.00 [-0.84, 0.84]
Gloeckl et al., 2012	-0.8	1.6	30	-0.6	1.4	30	13.0%	-0.20 [-0.96, 0.56]
Kaltsakas et al., 2017	0	0.6	12	1.1	0.8	12	16.9%	-1.10 [-1.67, -0.53]
Varga et al., 2007	-0.6	2.2	17	-0.7	2.6	22	5.3%	0.10 [-1.41, 1.61]
**Subtotal (95% CI)**			**87**			**96**	**46.9%**	**-0.42 [-1.05, 0.21]**
Heterogeneity: Tau² = 0.22; Chi² = 6.81, df = 3 (P = 0.08); I² = 56%								
Test for overall effect: Z = 1.30 (P = 0.20)								
**5.1.2 Poor to fair quality on PEDro scale**								
Ercin et al., 2020	-3.5	0.5	24	-2.6	0.3	23	24.1%	-0.90 [-1.13, -0.67]
Nasis et al., 2009	-0.3	1.9	21	-0.2	1.8	21	8.2%	-0.10 [-1.22, 1.02]
Vogiatzis et al., 2002	-1.9	0.6	18	-1.7	0.6	18	20.8%	-0.20 [-0.59, 0.19]
**Subtotal (95% CI)**			**63**			**62**	**53.1%**	**-0.49 [-1.08, 0.11]**
Heterogeneity: Tau² = 0.20; Chi² = 10.14, df = 2 (P = 0.006); I² = 80%								
Test for overall effect: Z = 1.61 (P = 0.11)								
**Total (95% CI)**			**150**			**158**	**100.0%**	**-0.47 [-0.86, -0.09]**
Heterogeneity: Tau² = 0.15; Chi² = 17.35, df = 6 (P = 0.008); I² = 65%								
Test for overall effect: Z = 2.40 (P = 0.02)								
Test for subgroup differences: Chi² = 0.03, df = 1 (P = 0.87), I² = 0%								

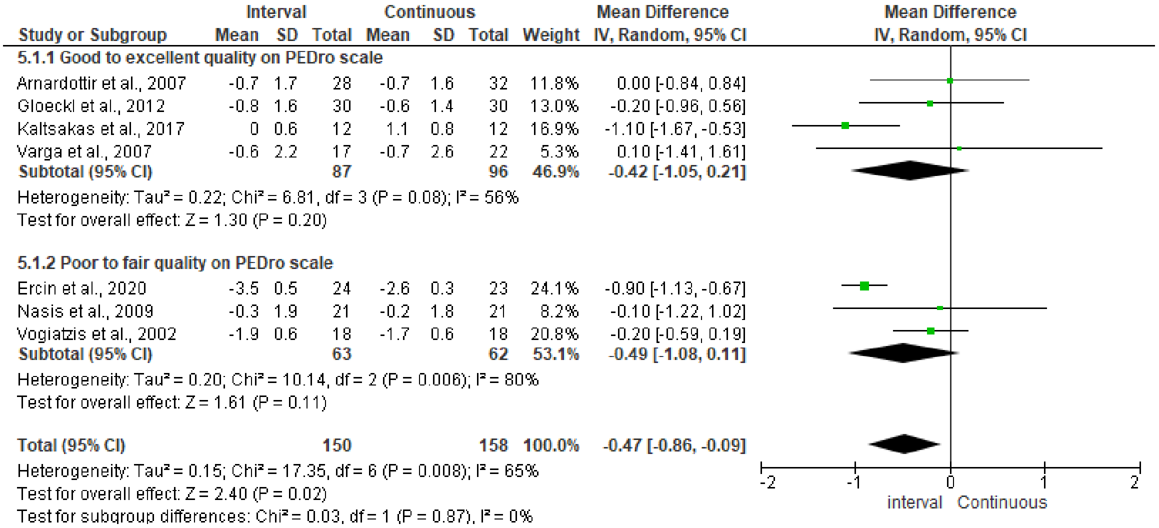

Figure S2.7. Forest plot of comparison between good to excellent quality studies and poor to fair quality studies rated on PEDro scale on leg discomfort (Borg's scale CR 0-10).

Study or Subgroup	Interval Mean	Interval SD	Interval Total	Continuous Mean	Continuous SD	Continuous Total	Weight	Mean Difference IV, Random, 95% CI
**6.1.1 Good to excellent quality on PEDro scale**								
Arnardottir et al., 2007	-0.3	1.4	28	-0.6	1.3	28	18.8%	0.30 [-0.41, 1.01]
Gloeckl et al., 2012	-0.9	1.9	30	-0.7	2.1	30	14.4%	-0.20 [-1.21, 0.81]
Kaltsakas et al., 2017	-1.3	0.1	12	0	0.6	12	24.3%	-1.30 [-1.64, -0.96]
Varga et al., 2007	-0.8	2.4	17	-0.3	3	22	7.8%	-0.50 [-2.20, 1.20]
**Subtotal (95% CI)**			**87**			**92**	**65.4%**	**-0.46 [-1.43, 0.52]**
Heterogeneity: Tau² = 0.75; Chi² = 18.27, df = 3 (P = 0.0004); I² = 84%								
Test for overall effect: Z = 0.92 (P = 0.36)								
**6.1.2 Poor to fair quality on PEDro scale**								
Ercin et al., 2020	-3	0.5	24	-2.2	0.6	23	24.7%	-0.80 [-1.12, -0.48]
Nasis et al., 2009	0.3	1.4	21	-0.2	3	21	10.0%	0.50 [-0.92, 1.92]
**Subtotal (95% CI)**			**45**			**44**	**34.6%**	**-0.34 [-1.56, 0.88]**
Heterogeneity: Tau² = 0.57; Chi² = 3.08, df = 1 (P = 0.08); I² = 68%								
Test for overall effect: Z = 0.55 (P = 0.58)								
**Total (95% CI)**			**132**			**136**	**100.0%**	**-0.48 [-1.04, 0.09]**
Heterogeneity: Tau² = 0.31; Chi² = 22.01, df = 5 (P = 0.0005); I² = 77%								
Test for overall effect: Z = 1.65 (P = 0.10)								
Test for subgroup differences: Chi² = 0.02, df = 1 (P = 0.89), I² = 0%								

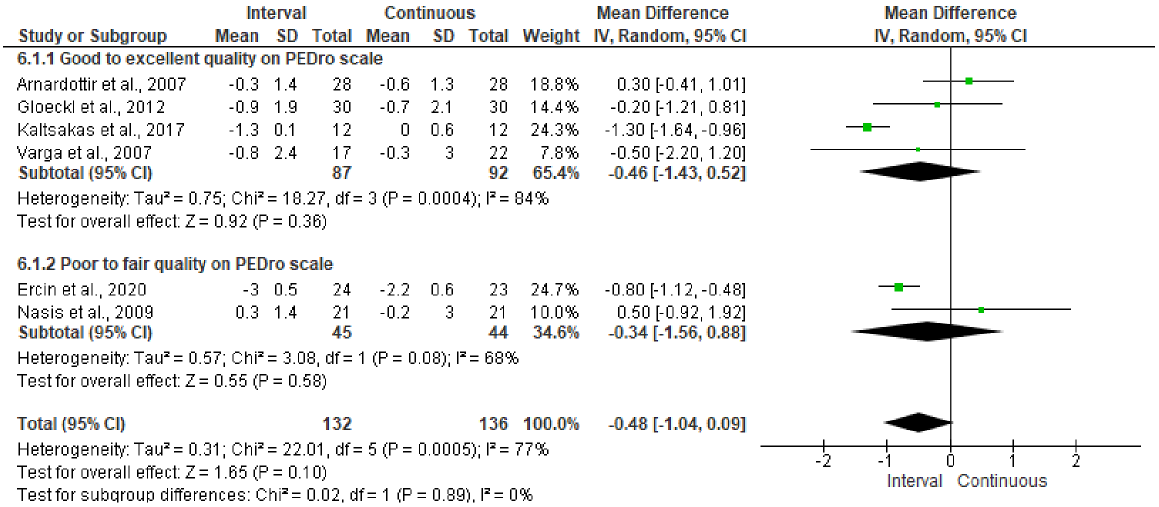

Analysis S3. Publication bias

Figure S3.1. Funnel plot illustration of the impact of publication bias on WR_{peak}.

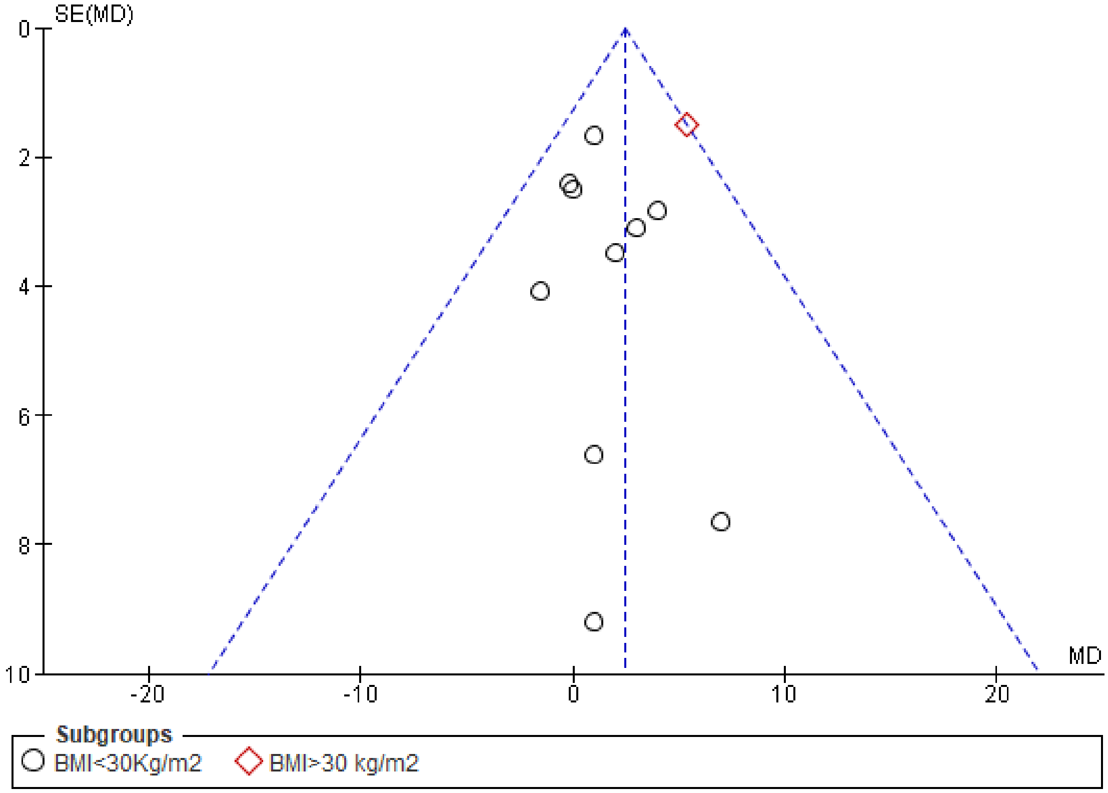

Supplement: sj-pdf-1-crd-10.1177_14799731211041506 – Supplemental Material for Effect of interval compared to continuous exercise training on physiological responses in patients with chronic respiratory diseases: A systematic review and meta-analysis [file sj-pdf-1-crd-10.1177_14799731211041506.pdf]
